# Validating the working alliance inventory in a mobile health smoking cessation program: A quasi-experimental study among Mexican adults who smoke

**DOI:** 10.18332/tid/213755

**Published:** 2026-01-13

**Authors:** Katia Gallegos-Carrillo, Paula Ramírez-Palacios, Arlette Chávez-Iñiguez, Ana Paula Cupertino, Francisco Cartujano-Barrera, Rosibel Rodríguez-Bolaños

**Affiliations:** 1 Epidemiological and Health Services Research Unit, Morelos, Mexican Social Security Institute, Cuernavaca, Mexico; 2 Center for Evaluation and Surveys Research, National Institute of Public Health, Cuernavaca, Mexico; 3 Department of Surgery, University of Rochester Medical Center, Rochester, United States; 4 Department of Public Health Sciences, University of Rochester Medical Center, Rochester, United States; 5 Center for Population Health Research, National Institute of Public Health, Cuernavaca, Mexico

**Keywords:** smoking cessation, counseling, mHealth, working alliance, mCessation

## Abstract

**INTRODUCTION:**

The therapeutic alliance has been identified as a key factor influencing smoking cessation success. This study aimed to evaluate the psychometric properties – internal consistency, factorial validity, and concurrent validity – of the Spanish version of the Working Alliance Inventory-Short (WAI-S) in a mobile health (mHealth) smoking cessation program for Mexican adults who smoke. Additionally, it examined the association between WAI-S score, smoking-related outcomes and program satisfaction.

**METHODS:**

A quasi-experimental study was conducted in Mexico between June and October 2021 with 100 adults who smoke intending to quit. The 12-week mHealth cessation program, grounded in Social Cognitive Theory, combined automated text messages and tailored counselor feedback. At program completion, 80 participants completed the 12-item WAI-S. Psychometric analyses included exploratory factor analysis, Kaiser-Meyer-Olkin and Bartlett’s tests, Cronbach’s alpha, and item-total correlations. Logistic regression models assessed the association of WAI-S scores with program satisfaction and smoking cessation outcomes.

**RESULTS:**

Exploratory factor analysis (n=80) revealed that two negatively worded items weakened internal consistency; their removal produced a refined 10-item scale with a robust two-factor structure and excellent reliability (Cronbach’s α=0.91). Higher reported therapeutic alliance scores were associated with greater odds of satisfaction with the overall program (AOR=1.12; 95% CI: 1.04–1.21), the digital application (AOR=1.10; 95% CI: 1.02–1.19), and the text message content (AOR=1.10; 95% CI: 1.03–1.18), compared with participants reporting lower alliance scores. No significant association emerged between WAI-S scores and self-reported or biochemically verified smoking abstinence.

**CONCLUSIONS:**

The 10-item Spanish WAI-S demonstrated strong psychometric validity for evaluating therapeutic alliance in an mHealth smoking cessation among Mexican adults. While not predictive of abstinence, higher alliance score correlated with greater satisfaction, underscoring the instrument’s potential for monitoring engagement and informing the design of more effective digital cessation programs.

## INTRODUCTION

Smoking is the leading cause of preventable morbidity and mortality worldwide. For health systems, it leads to excessive healthcare expenditures and, as a consequence, loss of work productivity^1^. Approximately 68% of individuals who smoke in high-income countries (HICs) want to quit smoking, and just over half attempt to quit each year^2^. One of the most effective ways is counseling or behavioral support, which increases the probability of quitting by 40% to 60% compared to minimal or no assistance^3^. A key factor in the success of counseling is the interaction between the therapist and the patient, characterized by trust, empathy, and effective communication^4^.

Therapeutic alliance refers to the quality of the relationship between the client and counselor, encompassing shared goals, cooperation, mutual trust, confidence, and positive regard^5^. In HICs, the therapeutic alliance has been a focus of research for over four decades and is recognized as one of the most robust predictors of treatment outcomes in psychotherapy and health interventions^6^. However, in low- and middle-income countries (LMICs) like Mexico, there is not enough evidence regarding the use of this construct to evaluate mobile phone-based smoking cessation.

Several models have been developed to assess the therapeutic alliance. Among them, the alliance model of Bordin^7^ is widely accepted across theoretical orientations and has served as the foundation for instruments measuring this construct^7,8^. The original Working Alliance Inventory (WAI) by Horvath and Greenberg^5^ included 36 items organized into three subscales: Bonds, Goals, and Tasks, with 12 items each. Responses are provided on a seven-point Likert scale, with higher scores indicating stronger alliance and better clinical outcomes^9^.

With the shift from face-to-face to digital interventions, growing evidence supports the effectiveness of internet-based programs in treating psychological disorders^10^. Consequently, adapted versions of the WAI, such as the WAI-S and WAI-SF, were developed for use in technology-supported interventions. Previous studies suggest that alliance scores are similar between internet-based and face-to-face therapies^11,12^.

In smoking cessation, the therapeutic alliance reflects tobacco users’ perceptions of the strengths and weaknesses of interventions, particularly regarding the bond between the patient who smoked, and the therapist or health team. The therapeutic alliance has been studied as a predictor of quit attempts^13^. These findings highlight the therapeutic alliance as a key factor in face-to-face cessation efforts^9^, but little is known about its role in mHealth, especially in LMICs.

Evidence from HICs indicates that mHealth program can effectively support smoking cessation^14,15^. These programs offer interactive, cost-effective treatments that can reach large populations^16,17^. Studies suggest that simulating the therapeutic alliance within mHealth programs may enhance motivation, engagement, and behavior change^18,19^. Holder et al.^19^ conclude that by simulating the working alliance, motivation, interactivity and individual adaptation, the effectiveness of fully automated mHealth programs for changing behaviors could be increased^19^. However, prior research has not focused on psychometric evaluation of alliance measures in this context.

This study aimed to assess the psychometric properties including internal consistency, factorial validity, and concurrent validity, of the Working Alliance Inventory-Short (WAI-S) in ‘Decídetexto’, a mobile-based smoking cessation program, to Mexican adults who smoke. The three-factor model of Bordin^7^ was tested in a fully remote intervention, and associations between WAI-S scores and smoking cessation-related outcomes were explored.

## METHODS

### Design and study population

This was a quasi-experimental study without a control group, conducted among 100 Mexican adults who smoke combustible cigarettes and expressed an intention to quit. The intervention, ‘Decídetexto-Mexico’, included three components: 1) A tablet-based software to collect smoking-related information and develop an individualized cessation plan; 2) A 12-week text messaging counseling program; and 3) Free provision of nicotine replacement therapy. ‘Decídetexto’ was based on Social Cognitive Theory. The text messaging component included: 1) pre-programmed daily messages (two to three per day); 2) automated responses to keyword-triggered texts (e.g. ‘desire’, ‘health’); and 3) ad hoc messages from a counselor, all delivered remotely over 12 weeks. Of the total participants enrolled in the program, 80 (80%) completed the 12-item Working Alliance Inventory–Short Form (WAI-S) at program completion, as well as both the pre- and post-intervention questionnaires. All measures were administered through the study’s digital platform immediately before and after the intervention period. The study took place between June and October 2021. Participants were recruited via online advertisements, social media, and clinical referrals as part of the ‘Decídetexto-Mexico’ program, more detailed procedures have been published elsewhere^20^.

### Ethics

The study protocol was approved by the Research Ethics Committee of participating institutions: National Institute of Public Health of Mexico (CI 1712) and the Mexican Social Security Institute (registration no. 2018-785-075). Before baseline surveys, eligible participants took part in Zoom video calls with research staff. During these calls, informed consent was obtained. A digital copy of the consent form was sent via SMS, and participants confirmed consent by replying with an SMS including their full name.

### Working Alliance Inventory Short Form (WAI-S)

At the end of the intervention, participants completed the 12-item Spanish version of the WAI-S^21,22^. Each item uses a seven-point Likert scale (1=never and 7=always). The WAI-S includes three subscales: 1) Goals: Agreement between patient and therapist on overall treatment goals; 2) Tasks: Agreement on tasks relevant to achieving these goals; and 3) Bonds: Emotional connection between patient and therapist, based on trust and mutual understanding. The WAI-S consists of 12 items rated from 1 (‘never’) to 7 (‘always’), yielding a total score between 12 and 84, with higher scores indicating a stronger therapeutic alliance. Two negatively worded items were reverse-coded before analysis.

### Satisfaction with ‘Decídetexto’

At program end, participants rated satisfaction with the digital application, text messages received, and the overall program. Responses were dichotomized as 0 (extremely unsatisfied, unsatisfied, or neutral) or 1 (satisfied or extremely satisfied).

### Smoking-related variables

At baseline, participants reported quit attempts in the prior 12 months (yes, no). At 12 weeks, they self-reported smoking abstinence (yes, no). Cigarettes per day were categorized as <10 or ≥10, based on self-report. Biochemical verification was conducted among those reporting abstinence: a urine cotinine test (<50 ng/mL = confirmed abstinence)^23,24^.

### Sociodemographic variables

Collected variables included gender (male, female), age (<50 vs ≥50 years), and education level (lower than college/college graduate studies).

### Statistical analysis

Descriptive statistics using mean and standard deviation (SD), and percentage (%), were obtained for all the main variables. Internal consistency of the WAI-S total and subscales was assessed using Cronbach’s alpha. Sampling adequacy was evaluated with the Kaiser-Meyer-Olkin (KMO) measure and Bartlett’s test of sphericity^25^. A principal components analysis (PCA)^26^ with varimax rotation (eigenvalues >1) identified underlying factors. Negatively worded items (4 and 10) were reverse-coded initially but removed from final analyses due to negative impact on scale validation. Item-test correlations were also assessed, with values below 0.30 indicating inadequate correlation with the overall scale^27,28^.

Logistic regression assessed associations between WAI-S total score and satisfaction outcomes. Models adjusted for gender, age, and education level. Associations between WAI-S score and smoking outcomes (self-reported abstinence, biochemically confirmed abstinence, were also examined). All tests were two-tailed, and p<0.05 was considered statistically significant. Analyses were performed using Stata Statistical Software: Release 15. StataCorp LLC, College Station, TX, USA.

## RESULTS

Participants had a mean age of 48.3 ± 11.6 years, and 40% reported smoking at least 10 cigarettes per day at baseline. At follow-up, 51.3% self-reported smoking cessation, and 30.0% achieved biochemically confirmed abstinence (Table 1).

**Table 1:** Follow-up characteristics of participants in ‘Decídetexto’ program, Mexico, 2021 (N=80)

*Characteristics*	*%*
**Age** (years) at baseline, mean (SD)	48.3 (11.6)
**Female**	51.2
**Education level**	
Lower than college	51.3
College graduate studies	48.7
**Cigarettes per day**	
<10	60.0
≥10	40.0
Severity of Dependence Scale, mean (SD)	9.36 (2.68)
Self-report cessation	51.3
Verified cessation	30.0

Table 2 shows that participants reported higher mean scores for the Tasks subscale (mean=6.52 ± 0.88) compared to the Goals (5.59 ± 1.28) and Bonds (6.11 ± 1.53) subscales. The Tasks subscale also showed stronger item-rest correlations, except for item 4 and item 10 (from the Goals subscale) and item 3 (from the Bonds subscale), which refer to agreement and perceived program helpfulness. Cronbach’s alpha values were 0.85 for Tasks, 0.49 for Goals, and 0.84 for Bonds. The overall WAI-S had a Cronbach’s alpha of 0.88. Excluding items 4 and 10 improved internal consistency, with Cronbach’s alpha increasing to 0.91 (Table 3).

**Table 2:** Descriptive data and global and subscales internal consistency of the Spanish version of the Working Alliance Inventory-Short, Mexico, 2021 (N=80)

*Items*	*Mean*	*SD*	*Median*	*Minimum*	*Maximum*	*Item-test correlation*	*Item-rest correlation*	*Average interitem correlation*	*Cronbach´s alpha*
**Subscale Tasks**									
1. The smoking cessation program and I agree about the things I will need to do to help improve my situation	6.53	1.19	7	1	7	0.6811	0.6027	0.3674	0.8646
2. What the smoking cessation program and I address gives me new ways of looking at my problem	6.00	1.61	7	1	7	0.7343	0.6662	0.3598	0.8608
8. My smoking cessation program and I agree on what is important for me to work on	6.46	1.31	7	0	7	0.7968	0.742	0.3509	0.8561
12. I believe the way we are working with my problem is correct	6.30	1.36	7	1	7	0.8345	0.7885	0.3456	0.8531
**Total score Tasks**	6.52	0.88	6.75	1.25	7				0.8476
**Subscale Goals**									
4. My smoking cessation program does not take into account what I am trying to accomplish	5.26	2.45	7	1	7	0.2626	0.1381	0.4268	0.8912
6. My smoking cessation program and I are working towards mutually agreed upon goals	6.50	1.19	7	1	7	0.8527	0.8112	0.343	0.8517
10. My smoking cessation program and I have different ideas on what my problems are	4.48	2.65	6	0	7	0.2684	0.1443	0.426	0.8909
11. My smoking cessation program and I understand the kind of changes that would be good for me	6.11	1.46	7	0	7	0.5295	0.4278	0.3889	0.875
**Total score Goals**	5.59	1.28	5.75	1	7				0.4946
**Subscale Bonds**									
3. I feel liked in my smoking cessation program	5.71	2.4	7	0	7	0.5923	0.4993	0.3800	0.8708
5. I am confident in my smoking cessation program’s ability to help me	6.55	1.09	7	1	7	0.828	0.7804	0.3465	0.8536
7. I feel appreciated in my smoking cessation program	5.91	2.25	7	0	7	0.7051	0.6313	0.364	0.8629
9. I trust my smoking cessation program	6.25	1.63	7	0	7	0.7286	0.6593	0.3606	0.8612
**Total score Bonds**	6.11	1.53	6.88	1	7				0.8414
**Global score**	6.01	1.05	6.33	1.08	7				0.8765

Pearson’s correlation coefficients used.

**Table 3:** Internal consistency without items 4 and 10 of the Spanish versions of the Working Alliance Inventory-Short, Mexico, 2021 (N=80)

*Items*	*Item-test correlation*	*Item-rest correlation*	*Average inter-item correlation*	*Cronbach’s alpha if item deleted*
1. The smoking cessation program and I agree about the things I will need to do to help improve my situation	0.6991	0.6200	0.5177	0.9062
2. What the smoking cessation program and I address gives me new ways of looking at my problem	0.7441	0.6744	0.5084	0.9030
3. I feel liked in my smoking cessation program	0.6225	0.5295	0.5336	0.9115
5. I am confident in my smoking cessation program’s ability to help me	0.8106	0.7561	0.4946	0.8980
6. My smoking cessation program and I are working towards mutually agreed upon goals	0.8566	0.8138	0.4851	0.8945
7. I feel appreciated in my smoking cessation program	0.7534	0.6857	0.5065	0.9023
8. My smoking cessation program and I agree on what is important for me to work on	0.8298	0.7801	0.4906	0.8966
9. I trust my smoking cessation program	0.7426	0.6725	0.5087	0.9031
11. My smoking cessation program and I understand the kind of changes that would be good for me	0.5628	0.4605	0.546	0.9154
12. I believe the way we are working with my problem is correct	0.8427	0.7963	0.4880	0.8956
**Global score**				**0.9117**

Items 4 and 10 were removed due to their low item-test correlations and improvements in internal consistency reliability upon their removal. Pearson’s correlation coefficients used.

In the PCA, Bartlett’s test of sphericity was significant (p<0.001), and the KMO measure was 0.82, indicating sampling adequacy. The scree plot showed three factors with eigenvalues >1.0, explaining 52.5%, 32.4%, and 13.6% of the variance, respectively. Together, these factors accounted for 98.6% of the total variance. Items 2, 3, 5, 6, 7, 8, and 9 loaded onto Factor 1; items 1, 11, and 12 loaded onto Factor 2; and negatively worded items 4 and 10 loaded onto Factor 3 (not shown in tables).

Construct validity of the 10-item scale (following the removal of items 4 and 10) was evaluated. The KMO measure of sampling adequacy was 0.83, indicating suitability for factor analysis. The Bartlett’s test of sphericity was significant (χ^2^=531.88, df=45, p<0.001), confirming that the correlation matrix was factorable. Two factors were extracted, explaining 55.98% and 39.53% of the variance, respectively. Together, the two factors accounted for 95.51% of the total variance. Items 2, 3, 5, 6, 7, 8, and 9 loaded on Factor 1. Items 1, 11, and 12 loaded on Factor 2 (not shown in tables).

Of the participants, 87.5% expressed satisfaction with the overall smoking cessation mHealth program, 91.3% satisfaction with the digital application, and 82.5% satisfaction with the text messages sent to cell phone. Table 4 shows that higher WAI-S total scores were significantly associated with greater satisfaction across all program components. Adjusted odds ratio (AOR) for overall program satisfaction was 1.12 (95% CI: 1.04–1.21); for digital application 1.10 (95% CI: 1.02–1.19); and for text messages 1.10 (95% CI: 1.03–1.18). No significant association was found between WAI-S score and self-reported or biochemically confirmed abstinence.

**Table 4:** Working Alliance Inventory-Short association with outcome variables related to self-perceived satisfaction and variables related to smoking cessation at 12 weeks of follow-up, Mexico, 2021 (N=80)

*Variables*	*OR (95% CI)*	*AOR (95% CI)*
**Satisfaction with overall smoking cessation mHealth program**		
No ®	1	1
Yes	1.14 (1.06–1.22)**	1.12 (1.04–1.21)**
**Satisfaction with the digital application**		
No ®	1	1
Yes	1.11 (1.04–1.18)**	1.10 (1.02–1.19)*
**Satisfaction with the text messages sent to cell phone**		
No ®	1	1
Yes	1.11 (1.05–1.18)**	1.10 (1.03–1.18)**
**Smoking cessation**		
**Smoking abstinence confirmed**		
No ®	1	1
Yes	1.01 (0.97–1.06)	1.05 (0.99–1.11)
**Smoking abstinence self-reported**		
No ®	1	1
Yes	1.02 (0.98–1.06)	1.04 (0.99–1.09)

For each unit of change in OR, the therapeutic alliance increases. AOR: adjusted odds ratio; adjusted by gender, age and education level. No = extremely unsatisfied/unsatisfied/neutral; Yes = extremely satisfied/satisfied. Only 10 items included, items 4 and 10 that are stated in the negative were deleted.

* p<0.05.

** p<0.01. ® Reference categories.

## DISCUSSION

The instrument employed in this study demonstrates strong reliability for evaluating the therapeutic alliance in smoking cessation^29^. The original WAI-S included two negatively phrased items intended to reduce response bias and increase respondent attentiveness^21^. However, the total scale showed higher reliability when these items were excluded. The removal of items 4 (‘My smoking cessation program doesn’t take into account what I’m trying to accomplish’) and 10 (‘My smoking cessation program and I have different ideas about my problems’) was supported by low item–total correlations and improved internal consistency. These items, which addressed relational disagreement, were embedded among predominantly positively worded statements, likely creating confusion or measurement error and thereby reducing their psychometric value.

Even so, the exploratory factor analysis revealed a clear two-factor structure of the WAI-S in the Mexican population. Bartlett’s test of sphericity indicated that the correlation matrix was favorable for factor analysis, supporting the adequacy of the data. Furthermore, the internal consistency of the instrument improved after removing two items with item–test correlations below 0.30, resulting in a robust 10-item version. This refined structure not only demonstrates strong psychometric properties but also captures the multidimensional nature of therapeutic adherence, encompassing both behavioral compliance and attitudinal aspects toward treatment.

A systematic review of the WAI-S measurement properties revealed inconsistent findings related to internal consistency, content validity, and cross-cultural applicability. Despite its utility in certain settings, concerns regarding item clarity and cultural interpretation suggest the need for refinement to more accurately capture interpersonal dynamics, such as shared decision-making and rapport, while reducing the influence of social desirability^29^.

This intervention was implemented during the COVID-19 pandemic^30^, when conducting the program entirely online posed significant challenges. Internet-based interventions create a different type of therapeutic bond, as they rely on text and technology rather than direct interpersonal interaction. Despite initial concerns, a previous study demonstrated that participants were able to establish meaningful therapeutic relationships through online platforms^31^.

In our study, we made minor modifications to the Working Alliance Inventory for guided internet interventions (WAI-I), mainly by replacing the term ‘therapist’ with ‘virtual environment’. We recognize that a more recent version, the WAI-Tech, is now available. Although our intervention was fully online, we chose to use the WAI-S to assess the therapeutic alliance. Other studies^12,32^ have applied the WAI to both face-to-face and internet-based interventions, or blended approaches combining both.

Developing and validating improved Spanish-language measures of the therapeutic alliance remains a priority. The mean alliance score in our study was slightly lower than that reported in other studies with Latino participants^33^. This finding supports previous evidence that text messaging can foster a strong therapeutic connection in smoking cessation programs.

Our study found that higher alliance scores were linked to greater satisfaction across components of the program. This highlights the importance of assessing satisfaction comprehensively and considering the contribution of individual program components.

### Limitations

This study has several limitations, we did not measure engagement intensity; adherence was defined solely by program completion, including self-reported smoking data that may introduce misclassification bias, the absence of a control group limiting causal inference, and potential residual confounding. Given that participants were recruited via digital platforms, there is a potential recruitment bias related to socioeconomic status and access to technology. Furthermore, varying levels of digital literacy may have influenced participants’ ability to fully engage with the intervention, potentially affecting both alliance perceptions and outcome reporting. Finally, findings may not be generalizable beyond the Mexican adult population enrolled in the ‘Decídetexto’ program. Our completion rate was comparable to or higher than rates reported in other text-based cessation interventions^34^.

## CONCLUSIONS

This study demonstrated that the Spanish version of the WAI-S is a valid tool for assessing the therapeutic alliance in a mobile smoking cessation program. Participants reported high satisfaction across all components of the intervention.

The results of the exploratory factor analysis suggest that the Spanish WAI-S is suitable for use in digital contexts; however, items 4 and 10, originally formulated with negative wording, may have caused confusion in a remote setting. We recommend revising these items or developing new ones to ensure that each subscale contains an equal number of positively worded statements. Future studies should incorporate a control group and a larger sample size to enable stratified analyses by tobacco use intensity and frequency. Moreover, randomized controlled and cross-cultural validation studies are warranted to strengthen the evidence base before advancing specific programmatic recommendations.

## Data Availability

The data supporting this research are available from the authors on reasonable request.
